# Fault Diagnosis of Rolling Bearings Using Denoising Multi-Channel Mixture of CNN and Mamba-Enhanced Adaptive Self-Attention LSTM

**DOI:** 10.3390/s25216652

**Published:** 2025-10-31

**Authors:** Songjiang Lai, Tsun-Hin Cheung, Ka-Chun Fung, Kaiwen Xue, Jiayi Zhao, Hana Lebeta Goshu, Zihang Lyu, Kin-Man Lam

**Affiliations:** 1 Department of Electrical and Electronic Engineering, The Hong Kong Polytechnic University, Kowloon, Hong Kong; song-jiang.lai@connect.polyu.hk (S.L.); tsun-hin.cheung@connect.polyu.hk (T.-H.C.); jear123.xue@connect.polyu.hk (K.X.); hana-lebeta.goshu@connect.polyu.hk (H.L.G.); zihang.lyu@connect.polyu.hk (Z.L.); 2 Centre for Advances in Reliability and Safety, New Territories, Hong Kong; xiaona.zhao@connect.polyu.hk

**Keywords:** rolling bearings, fault diagnosis, multi-channel denoising, mixture of experts, mamba-enhanced algorithms, self-attention mechanism, long short-term memory (LSTM)

## Abstract

Recent advancements in deep learning have significantly improved fault diagnosis methods. However, challenges such as insufficient feature extraction, limited long-range dependency modeling, and environmental noise continue to hinder their effectiveness. This paper presents a novel mixture of multi-view convolutional (MOM-Conv) layers integrating the Mixture of Experts (MOE) mechanism. This design effectively captures and fuses both local and contextual information, thereby enhancing feature extraction and representation. This proposed approach aims to improve prediction accuracy under varying noise conditions, particularly in rolling ball bearing systems characterized by noisy signals. Additionally, we propose the Mamba-enhanced adaptive self-attention long short-term memory (MASA-LSTM) model, which effectively captures both global and local dependencies in ultra-long time series data. This model addresses the limitations of traditional models in extracting long-range dependencies from such signals. The architecture also integrates a multi-step temporal state fusion mechanism to optimize information flow and incorporates adaptive parameter tuning, thereby improving dynamic adaptability within the LSTM framework. To further mitigate the impact of noise, we transform vibration signals into denoised multi-channel representations, enhancing model stability in noisy environments. Experimental results show that our proposed model outperforms existing state-of-the-art approaches on both the Paderborn and Case Western Reserve University bearing datasets, demonstrating remarkable robustness and effectiveness across various noise levels.

## 1. Introduction

In modern industry, it is crucial to monitor the status of equipment and identify operational failures for ensuring reliability and safety. Rolling bearings are among the key components that often indicate low reliability in technical systems. They play a vital role across various industrial sectors, including aerospace, automotive, marine, and others, serving as an essential component of rotating machinery [1,2,3]. Unanticipated bearing failures pose significant risks to human safety and can result in substantial economic losses [4,5]. Empirical data shows that almost 50% of all bearing failures are due to insufficient lubrication, which typically results in increased temperature and vibration inside the bearings [6]. Recently, the identification of bearing-related issues has increased, driven by advancements in intelligent diagnostic methodologies [7,8]. Bearing faults can severely impact the reliability and stability of complex mechanical systems, resulting in substantial financial losses and safety concerns [9,10]. Hence, it is imperative to develop efficient methods for bearing fault diagnosis.

Fault diagnosis approaches for bearings include signal analysis-based methods, model-based methods, and data-driven methods. Among these, data-driven methods, particularly those based on machine learning and deep learning [11], are the focus of current research. Compared to model-based and signal analysis-based methods, data-driven approaches require less domain-specific expertise. Conventional machine learning methods usually consist of two stages: feature extraction using various signal processing methods, followed by fault pattern classification using shallow algorithms, such as backpropagation neural network (BPNN) [12], k-nearest neighbor (k-NN) [13], and support vector machines (SVMs) [14]. However, these algorithms often struggle to effectively represent complex feature information and heavily rely on the quality of manually selected features [15]. On the other hand, machine learning methods that combine with traditional physics-based approaches demonstrate stronger generalization capabilities and greater application potential. For instance, Sobie et al. [16] proposed a simulation-driven machine learning method for bearing fault diagnosis, utilizing simulated data and validating it against experimental datasets. Matania et al. [17] introduced a hybrid algorithm for classifying bearing spall types using zero-fault-shot learning, integrating physics-based methods with machine learning to achieve accurate classification without relying on faulty data. However, such approaches suffer from significant data dependency, and the incorporation of physics-based methods often increases model complexity, thereby reducing interpretability. In contrast, deep learning (DL) models leverage large datasets and complex network structures to learn diverse feature representations, enabling superior diagnostic performance compared to traditional machine learning methods [18,19]. For instance, Ye and Yu [20] applied a deep morphological Convolutional Neural Network (CNN) to analyze vibration signals, significantly improving fault diagnosis precision. Similarly, Wang et al. [21] proposed a 1D-CNN model that extracts features directly from raw sensor signals, specifically designed for diagnosing faults in stationary-shaft and planetary gearboxes. Wang et al. [22] developed 1D-CNN-LSTM to capture features from multi-sensor vibration data, incorporating feature fusion to improve bearing fault diagnostic precision. Chen et al. [23] introduced a transfer learning approach using a flexible CNN-LSTM architecture, enabling accurate fault diagnosis across various operational conditions. Additionally, Chen et al. [24] proposed a fault diagnosis model integrating multi-scale CNN-LSTM with residual modules. This model employs convolutional kernels of different sizes to enhance fault feature extraction across different frequency ranges.

To address the issue of reduced fault diagnosis accuracy caused by ambient noise interference, researchers have enhanced the noise resilience of diagnostic models through model optimization and feature fusion. Zhang et al. [25] proposed a CNN architecture called TICNN, which incorporates data augmentation and training interference resistance techniques. This model achieves high diagnostic accuracy without the need for data denoising. Qiao et al. [26] used single-signal dimension maps and time-frequency maps as model inputs, sequentially applying CNNs and LSTM networks to extract complementary spatial and temporal features. The model achieves enhanced noise resistance capability. Zhou et al. [27] introduced an approach that combines a broad convolution-kernel CNN with frequency domain information, achieving high accuracy even under noisy conditions. Furthermore, Zou et al. [28] proposed a CNN-based denoising method that employs an adversarial learning strategy to improve the quality of noisy samples, thereby enhancing robustness and classification accuracy in fault diagnosis tasks.

In practice, the effectiveness of fault diagnosis algorithms primarily relies on the availability of sufficient labeled data for model training, which is a challenging requirement to meet in practical applications. To address data scarcity, Cao et al. [29] proposed AdvSGAN, an adversarial signal generation network based on Generative Adversarial Networks (GANs), which generates extra training data to improve diagnostic precision and model robustness. Furthermore, Xu et al. [30] introduced a novel data fusion approach that integrates a bearing dynamic model with a multi-agent diverse GAN (MAD-GAN) to simulate and rectify fault data, effectively mitigating data scarcity challenges in intelligent fault diagnosis. Transfer learning (TL) has also gained significant attention as a solution to data insufficiency [2]. Shao et al. [31] employed transfer learning to utilize pretrained models for feature extraction from limited labeled time-frequency images, enhancing classification accuracy. Yang et al. [32] proposed a federated transfer learning model that allows data-scarce clients to leverage knowledge from data-rich clients through dynamic weighting based on Maximum Mean Discrepancy (MMD), thereby improving diagnostic accuracy.

Recent research has increasingly focused on developing models that enhance feature extraction and representation under limited data conditions. Xu et al. [33] proposed a CNN-LSTM hybrid model that combines conventional signal analysis with deep learning techniques for enhanced automated feature extraction. Wang et al. [34] introduced the 1D2D-EDL network, which utilizes a relative angle matrix to convert 1D vibration signals into 2D images, enhancing feature extraction and classification accuracy in data-scarcity scenarios. Tang et al. [35] developed a hybrid model integrating a quadratic neural network (QNN) with Bi-LSTM, enabling efficient and interpretable fault diagnosis of rolling bearings by enhancing both feature extraction and representation under limited data availability.

In summary, bearing fault diagnosis encounters several key challenges: (1) Insufficient capabilities in feature extraction and representation from bearing vibration signals, leading to the loss of critical information. (2) Noise interference, which significantly undermines the accuracy and robustness of diagnostic methods, particularly under complex operating conditions. (3) Limited ability to capture long-range dependencies in ultra-long sequences, especially in scenarios characterized by data scarcity.

To address these challenges, this study proposes a new approach for converting 1D raw signals into 2D spatial signals in the form of images, termed the multi-channel denoised image input method, and an ensemble deep learning framework composed of MOM-CNN and the MASA-LSTM Network. The proposed Mamba-enhanced adaptive self-attention LSTM integrates a self-attention mechanism and an external Mamba block to comprehensively capture both global and local long-range dependencies, improving the model’s ability to process ultra-long time series data with complex sequential patterns. To leverage previous time states and enable dynamic adjustments within the LSTM architecture, the model also incorporates a multi-step state fusion mechanism and adaptive parameter tuning. Furthermore, the multi-channel denoised image input methodology converts 1D vibration signals into a 2D structure, in the form of an image, with mean and median filtering applied to enhance robustness against noise. The discrete Fourier transform (DFT) is also utilized to distinguish signal from noise, further alleviating noise interference. The proposed MOM-CNN further improves feature extraction efficiency and optimizes feature representation by enhancing semantic feature extraction and fusion across regions at varying scales and filtering redundancy, thereby boosting model performance and reducing noise impact.

The main contributions of this research are outlined below:

- A novel method that transforms 1D vibration signals into denoised multi-channel images is proposed. This enhances input representation by capturing a broader spectrum of features, improving the model’s robustness against noise and enabling more accurate fault diagnosis in complex environments.
- The proposed MOM-Conv layer enhances feature extraction and representation capabilities by integrating multi-view feature extraction with MOE mechanism, allowing the model to adaptively emphasize relevant features while minimizing redundant information. This design significantly improves the model’s predictive performance under varying operational conditions.
- A novel MASA-LSTM model that captures global and local long-range dependencies in time series data is presented. By integrating an external Mamba block and a self-attention mechanism, the model enhances its ability to accurately process and analyze extensive sequences. Furthermore, a multi-step state fusion mechanism and adaptive parameter tuning further enhance information flow and dynamic adaptability.
- Finally, this paper explores the utility and performance of the proposed hybrid network, which integrates the multi-channel denoised image input method, MOM-CN, and MASA-LSTM. This analysis provides valuable insights into the effectiveness of the ensemble architecture for bearing fault diagnosis.

This paper is structured as follows: Section 2 details the components of the proposed method and relevant theoretical foundation, including the MOM-Conv layer, the multi-channel denoised image input methodology, and the MASA-LSTM network. In Section 3, experimental results are shown to evaluate the proposed method and compare it with state-of-the-art methods. Finally, Section 4 concludes the paper.

## 2. Theory and Methodology

### 2.1. Overall Framework

In this paper, we introduce a novel hybrid network for identifying faults in rolling bearings. This network combines a mixture of multi-view CNN with a unique Mamba-enhanced adaptive self-attention LSTM network and takes multi-channel denoised signals in the form of images as input. This section offers a comprehensive explanation of our proposed method, which comprises these three key components.

**Figure 1:**
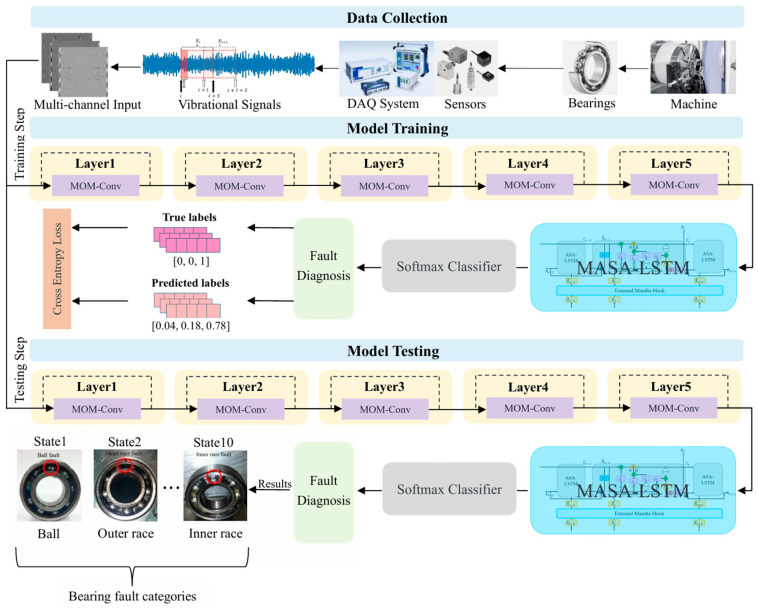
The framework of the proposed model.

Figure 1 shows the structure of the proposed fault diagnosis framework. The multi-channel denoised image input methodology is initially employed to transform one-dimensional vibration signals into two-dimensional images, which are then fed into our proposed hybrid network. This hybrid network consists of three primary components: a 5-layer Mixture of Multi-view convolutional neural network (MOM-CNN), a Mamba-enhanced Adaptive Self-Attention LSTM (MASA-LSTM), and a final SoftMax classifier. Each MOM-Conv layer performs a series of convolutional operations aimed at extracting sufficient features. This includes one standard convolution and three dilated convolutions. Additionally, a Mixture of Experts (MoE) mechanism is incorporated to enhance the integration of semantic features across different scales. Following this, global average pooling and a 2-layer fully connected network are employed to further aggregate global feature information. Within the MASA-LSTM, the Mamba block and self-attention mechanism are designed to assist the LSTM in more effectively learning both global and local long-range dependencies. Additionally, a multi-step state fusion mechanism combined with adaptive parameter tuning further improves information flow and dynamic adaptability. Finally, the output of MASA-LSTM is sent into the SoftMax classifier composed of multilayer perceptrons and a SoftMax function for the ultimate fault diagnosis.

The entire diagnosis process is segmented into three stages: collecting and processing data, training the model, and testing the model. The multi-channel images are created during the data collection stage, then multi-channel training images are fed to the model for training. Finally, in the testing stage, the performance of the trained model for fault diagnosis is evaluated.

### 2.2. Multi-Channel Denoised Image Input Methodology

To analyze the status of ball bearings, 1024 consecutive data points are selected from the original vibration signal in utilized datasets to form a sample. Samples are collected at random positions of the vibration signal and are denoted as $X_{i}=\left\{x_{n_{i}},\;x_{n_{i+1}},\;\ldots ,\;x_{n_{i}+1023}\right\}$, where $x_{n_{i}}$ represents the data point at the $n_{i}$-th position of the signal and $n_{i}$ is the starting position of the data points selected for the sample $X_{i}$. Usually, vibration signals collected in complex environments are severely contaminated by noise, which significantly degrades the accuracy of prediction models. To address this issue, we propose a novel preprocessing method, which transforms the 1-D input sequence into multiple channels, which are then represented in the frequency domain using discrete Fourier transform (DFT). The vibration signals used in our experiments are sourced from two datasets, both of which contain noisy signals captured under real-world scenarios.

To guide the model to learn robustly, denoised versions of $X_{i}$ are fused to form the input to our fault-diagnosis model. To better distinguish the clean and noise components of the input, its frequency representation, which is computed by using 2D-DFT, is employed. Mean and median filtering are applied to vibration signals, and the corresponding processed signals are denoted as ${\bar{x}}_{i}$ and ${\tilde{x}}_{i}$. Assume that a vibration signal contains L data points, i.e., its length is L. To predict the status of ball bearings at the time instance $n_{i}$, a window of size S=1024 is used to form a sample $X_{i}=\left\{x_{n_{i}},\;x_{n_{i+1}},\;\ldots ,\;x_{n_{i}+S-1}\;\right\}\in R^{1\times S}$, where $n_{i}\;\in \;[0,\;L\;-\;S]$. The corresponding mean and median samples are denoted as ${\bar{X}}_{i}$ and ${\tilde{X}}_{i}$. The mean and median of the data point $x_{i}$ are both computed based on window sizes of 10, with $x_{i}$ at the center of the corresponding windows. Padding zero is employed if there are insufficient data points for computing the mean and median of a data point. The corresponding mean and median data points are denoted as ${\bar{x}}_{i}$ and ${\tilde{x}}_{i}$. $X_{i}$, ${\bar{X}}_{i}$, and ${\tilde{X}}_{i}$ are combined to form $F_{i}={\left(X_{i}\;{\bar{X}}_{i}\;{\tilde{X}}_{i}\right)}^{T}\in R^{3\times S}$, which is then transformed into the frequency domain. In the frequency domain, noise can be better separated from the original signal, so the noise effect can be greatly reduced, thereby improving the prediction accuracy of our model.

To better perform vibration analysis, the input X is transformed into frequency domain using 2D-FFT. This can not only study the frequency content of the three input channels, but also the correlation between them. Vibration analysis is one of the most effective techniques for diagnosing ball-bearing faults like unbalanced shafts, misalignments, bearing wear, and gear faults in mechanical systems. In the frequency spectrum, patterns related to mechanical faults can be more easily identified. Mechanical faults often produce periodic vibration patterns that are difficult to interpret in the time domain but can be easily identifiable in the frequency domain. When a system has a fault, it generates vibrations at specific frequencies related to the fault’s nature. These frequencies often correspond to rotational or structural resonances. The 2D-DFT of the combined vibration signal $F_{i}(m,n)$ is given by

(1) $$F_{i}^{\prime }=\sum_{m=0}^{3-1}\sum_{n=0}^{S-1}F_{i}(m,n)e^{-j2\pi (\frac{k_{1}m}{3}+\frac{k_{2}n}{S})}$$

where $F_{i}(m,n)$ represents the value at row m (channel index) and column n (current data point in signal i) of the 2D matrix formed by $X_{i}$, ${\bar{X}}_{i}$, and ${\tilde{X}}_{i}$. S is the length of each 1D vibration signal channel (number of columns) and the total number of vibration signal channels (number of rows) is 3. $k_{1}$ and $k_{2}$ are the frequency indices corresponding to the rows and columns. The permissible range for $k_{1}$ extends from 0 to 2, while the range for $k_{2}$ spans from 0 to S−1. After the transformation, each vibration input is converted into frequency domain, facilitating fault analysis. After applying FFT to the combined input vibration signal $F\in R^{3\times S}$, the transformation output is complex, denoted as $F^{\prime }\in R^{6\times S}$. It is worth noting that $F^{\prime }$ contains a real component and an imaginary component, denoted as $F_{real}^{\prime }$ and $F_{imag}^{\prime }$, respectively. In other words, the transformed input contains two vibration-signal maps, $F_{real}^{\prime }\in R^{3\times S}$ and $F_{imag}^{\prime }\in R^{3\times S}$.

**Figure 2:**
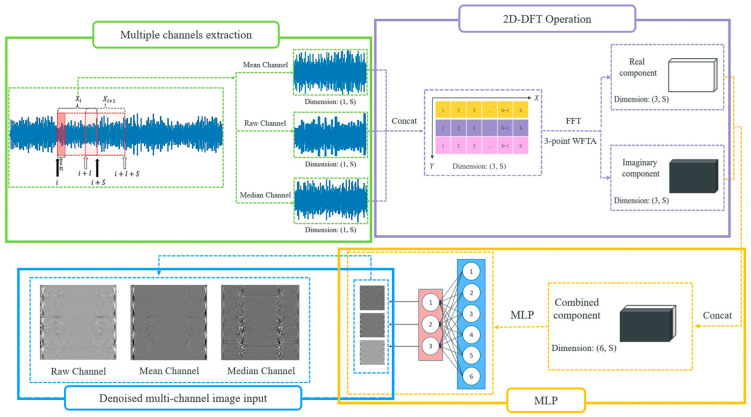
The workflow of generating multi-channel denoised images.

**Figure 3:**
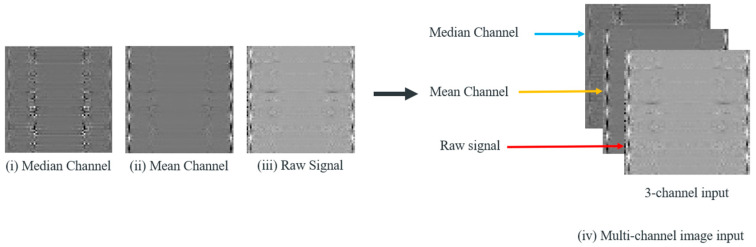
The process of transforming the vibration data points into multi-channel images.

These components are then concatenated and sent through a multilayer perceptron for fusing and generating the original number of channels. This output is denoted as $G_{i}\in R^{3\times S}$. For the last step, list the data of each row in the former generated 2D frequency map by row and column, respectively, then transform them into three-channel 2D-dimentional tensors and a multi-channel denoised image sample is finally generated. The overall operation flow of generating one multi-channel denoised image sample is illustrated in Figure 2. The generated 2D images highlight key texture features and fault characteristics more effectively than raw 1D signals. The spatial arrangement of frequency components in 2D images allows models to capture subtle differences in signal patterns, such as repetitive anomalies and texture changes, which are indicative of different bearing conditions. These features are harder to detect in 1D signals. While 1D signals primarily capture temporal patterns, 2D representations contain both temporal and frequency information, revealing richer patterns associated with faults. Additionally, by transforming 1D signals into 2D images, the differences between different fault types of rolling bearings can be amplified. This conversion can lead to create clear separations between different fault classes based on unique patterns in the temporal-spectral domain. This improves the model’s ability to distinguish between various bearing health conditions, leading to more accurate diagnosis. The generated multi-channel denoised image sample is illustrated in Figure 3 and there exists a relationship between S and the width W and height H of the image for each channel, as shown in Equation (2). Experiments demonstrate that as the number of channels grows, the accuracy also increases.

(2) S=W×H

In addition, in order to improve the effectiveness of training the model, each sample is normalized to have zero mean and unit variance prior to the training process. This strategy is appropriate for data distributions with imprecise limits and potential outliers.

### 2.3. Mixture of Multi-View Convolutional Layer

The efficient and comprehensive extraction and representation of features from bearing vibration signals is crucial for fault diagnosis. Convolutional layers with a stride of 2 and a kernel size of 3 are widely utilized for feature extraction from bearing signals due to their simplicity and efficiency. However, such convolution operations often result in significant loss of spatial details, as their receptive fields are limited to specific local regions, thereby neglecting broader or even global contextual information. For the complex input images obtained through the multi-channel denoised image input method, even minor details can profoundly influence the model’s final determinations.

To address this issue, we propose the MOM-Conv, which aims to combine the multi-view feature extraction with the Mixture of Experts (MOE) mechanism. This integration creates a robust architecture that flexibly captures both local and contextual information. By capturing features across different scales of local perspectives, the model’s robustness to scale variations is enhanced. The MOE mechanism efficiently fuses these features and allocates varying weights to them, enabling adaptive emphasis on important features while diminishing the influence of less relevant ones, thereby reducing noise interference from the input. Furthermore, by extracting global information and integrating it with the fused features from MOE mechanism, the representation of features can be further reinforced. Consequently, the model benefits from a rich set of relevant and multi-view features, improving its ability to detect anomalies and providing a more comprehensive understanding of the bearing condition. Ultimately, this approach enhances the accuracy of fault diagnosis and improves performance in predictive maintenance applications.

**Figure 4:**
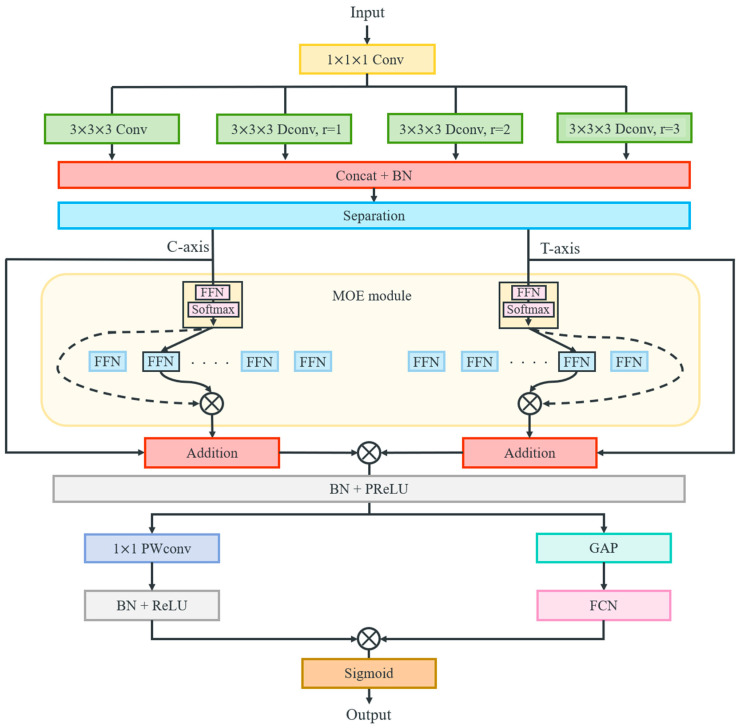
The architecture of the MOM-Conv layer.

The architecture of MOM-Conv layer is illustrated in Figure 4. This layer initially employs a convolutional operation with a stride of 2 and a kernel size of 3 for feature extraction. Concurrently, three dilated convolutions with a stride of 2, a kernel size of 3, and dilation factors of [1,2,3] are applied to capture feature information over broader spatial regions at varying scales. Then the resulting features are concatenated and normalized as follows:

(3) $${\tilde{G}}_{1}\left(X\right)={Conv3d}_{k=3,s=2,p=1}\left({Conv3d}_{k=1,s=1}(X)\right)$$

(4) $${\tilde{G}}_{2}(X)={DConv3d}_{k=3,s=2,p=2}^{r=1}\left({Conv3d}_{k=1,s=1}(X)\right)$$

(5) $${\tilde{G}}_{3}(X)={DConv3d}_{k=3,s=2,p=3}^{r=2}\left({Conv3d}_{k=1,s=1}(X)\right)$$

(6) $${\tilde{G}}_{4}(X)={DConv3d}_{k=3,s=2,p=4}^{r=3}\left({Conv3d}_{k=1,s=1}(X)\right)$$

(7) $${\tilde{G}}_{f}(X)=BN(Concat\left({\tilde{G}}_{1}\left(X\right),\;{\tilde{G}}_{2}\left(X\right),{\tilde{G}}_{3}\left(X\right),{\tilde{G}}_{4}\left(X\right)\right))$$

where $X\in R^{\left(b,c,t,\;h,\;w\right)}$ denotes the input features, ${\tilde{G}}_{1}\left(X\right)$ represents features extracted by the standard convolution from the immediate neighborhood, and ${\tilde{G}}_{2}\left(X\right)$, ${\tilde{G}}_{3}\left(X\right)$ and ${\tilde{G}}_{4}\left(X\right)$ correspond to the features extracted via the dilated convolution with specified dilation factors. Furthermore, k, s, p represent the kernel size, stride and padding, respectively. To enhance the fusion of features extracted from different regions, the Mixture of Experts (MOE) mechanism is employed to selectively amplify valuable information and filter out redundancy, thereby improving feature representation. The previously concatenated features are separated along channel and temporal dimensions, allowing for multi-perspective feature fusion.

Therefore, the features are separated along the channel and temporal dimension and flattened to $X_{c}\in R^{\left(b,c,h\times w\right)}$ and $X_{t}\in R^{\left(b,t,h\times w\right)}$, respectively. Then, these separated features are fed into the Mixture of Experts (MoE) mechanism [36]. In this mechanism, each expert $E_{i}\left(x\right)$ produces a feature transformation, while a gating network generates scores $z_{i}\left(x\right)$. The SoftMax-normalized gating weights $g_{i}\left(x\right)$ are used to select the top K experts, and the final output is a weighted sum of the selected experts’ outputs, as follows:

(8) $${\;E}_{i}\left(x\right)=W_{i}^{\prime }\cdot x+b_{i}^{\prime },\;z_{i}\left(x\right)=W_{i}\cdot x+b_{i}$$

(9) $$g_{i}\left(x\right)=\frac{\exp\left(z_{i}\left(x\right)\right)}{\sum_{j-1}^{n}exp(z_{j}\left(x\right))}$$

(10) $$I=TopK\left(\left\{g_{1}\left(x\right),\;g_{2}\left(x\right),\;\ldots ,\;g_{n}\left(x\right)\right\}\right)$$

(11) $$y=\sum_{i\in I}g_{i}\left(x\right)\cdot E_{i}\left(x\right)$$

where I stores the indices of the top K experts based on the gating weights $g_{i}(x)$. This allows the model to adaptively focus on the most relevant feature transformations. Thus, the outputs of the MOE mechanism are computed as follows:

(12) $$\left\{\begin{array}{c} Y_{c}=MOE\left(X_{c}\right)\\ Y_{t}=MOE\left(X_{t}\right) \end{array}\right.$$

where $Y_{c}$ and $Y_{c}$ represent the outputs of the MoE mechanism for the channel and temporal dimensions, respectively. A residual structure connects the input and output of the MOE to facilitate efficient gradient flow:

(13) $$Y_{c}^{\prime }=X_{c}+Y_{c},\;Y_{t}^{\prime }=X_{t}+Y_{t}$$

The features obtained from the channel and temporal perspectives are then reshaped as $Y_{c}^{\prime }\in R^{\left(b,c,1,\;h,w\right)}$ and $Y_{t}^{\prime }\in R^{\left(b,1,t,\;h,w\right)},$ respectively. These are then combined to produce the final output $Y^{\prime }\in R^{\left(b,c,t,\;h,w\right)}$, followed by batch normalization and the PReLU function are applied to ensure robust feature representation:

(14) $$Y^{\prime }=Y_{c}^{\prime }⊗Y_{t}^{\prime }$$

(15) $$Y=PReLU(BN\left(Y^{\prime }\right))$$

where ⊗  represents the element-wise multiplication of two feature maps. Subsequently, the features are processed through two branches: one branch employs global average pooling and 2-layer fully connected network, wherein the feature channels are initially compressed to a dimension of $\frac{C}{16}$ and then restored to c. This process effectively extracts global feature information while emphasizing the most informative regions:

(16) $$F_{g}=FCN\left(GAP\left(Y\right)\right)$$

The other branch utilizes point-wise convolutions along with normalization and the ReLU function to achieve further feature fusion and enhance feature representation:

(17) $$F_{c}=ReLU(BN\left(P{WConv}_{k=1,s=1}\left(Y\right)\right))$$

Finally, the features from both branches are merged and processed using the sigmoid function to obtain the optimized feature set:

(18) $$F_{out}={\delta (F}_{g}⊗F_{c})$$

where δ(∗)  represents the sigmoid function, thereby forming the MOM-Conv layer.

### 2.4. Mamba-Enhanced Adaptive Self-Attention LSTM Network

**Figure 5:**
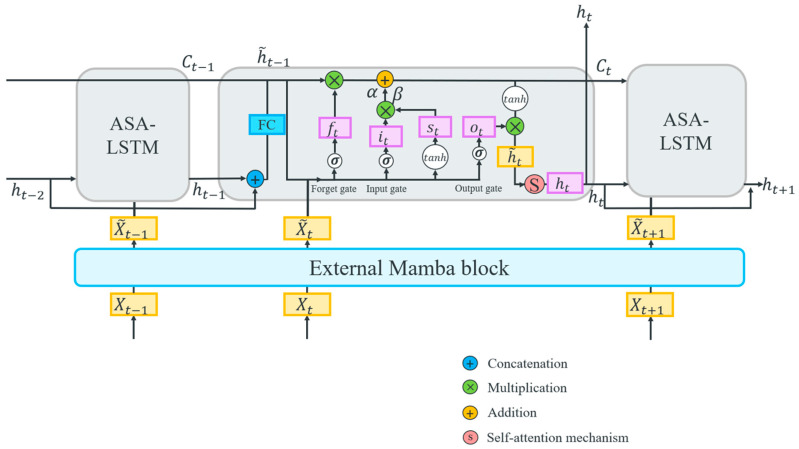
The structure of the MASA-LSTM network.

Although LSTM networks exhibit strong performance in capturing long-range dependencies within time series, making them a common choice as the backbone network for extracting such dependencies, they nonetheless face limitations in their ability to handle extremely long sequences because the interactions between features can become complex with many time steps, making it harder for the LSTM to identify long-range dependencies effectively. To address this limitation and further improve the model’s ability to extract long-range dependencies from an ultra-long time series signal, a Mamba-enhanced adaptive self-attention LSTM (MASA-LSTM) is proposed, as shown in Figure 5. The Mamba model and self-attention mechanism each have their respective strengths in capturing long-range dependencies in time series of varying lengths. Therefore, MASA-LSTM employs an external Mamba block to extract the global long-range dependencies of the input. Subsequently, within each time step iteration, we introduce a multi-head self-attention mechanism to capture local long-range dependencies. These enhancements boost the model’s capability to capture complex dependencies in time series data or the intrinsic correlations among features. Additionally, integrating trainable parameters into the LSTM gate equations increases the network’s flexibility in managing information flow, allowing for dynamic adjustments in forgetting, updating, and outputting processes. This adaptation maximizes the LSTM’s learning capability and improves noise resistance. Moreover, the update mechanism is restructured to utilize hidden states from the two preceding time steps, thereby strengthening the model’s capacity to capture complex dependencies and temporal patterns.

**Figure 6:**
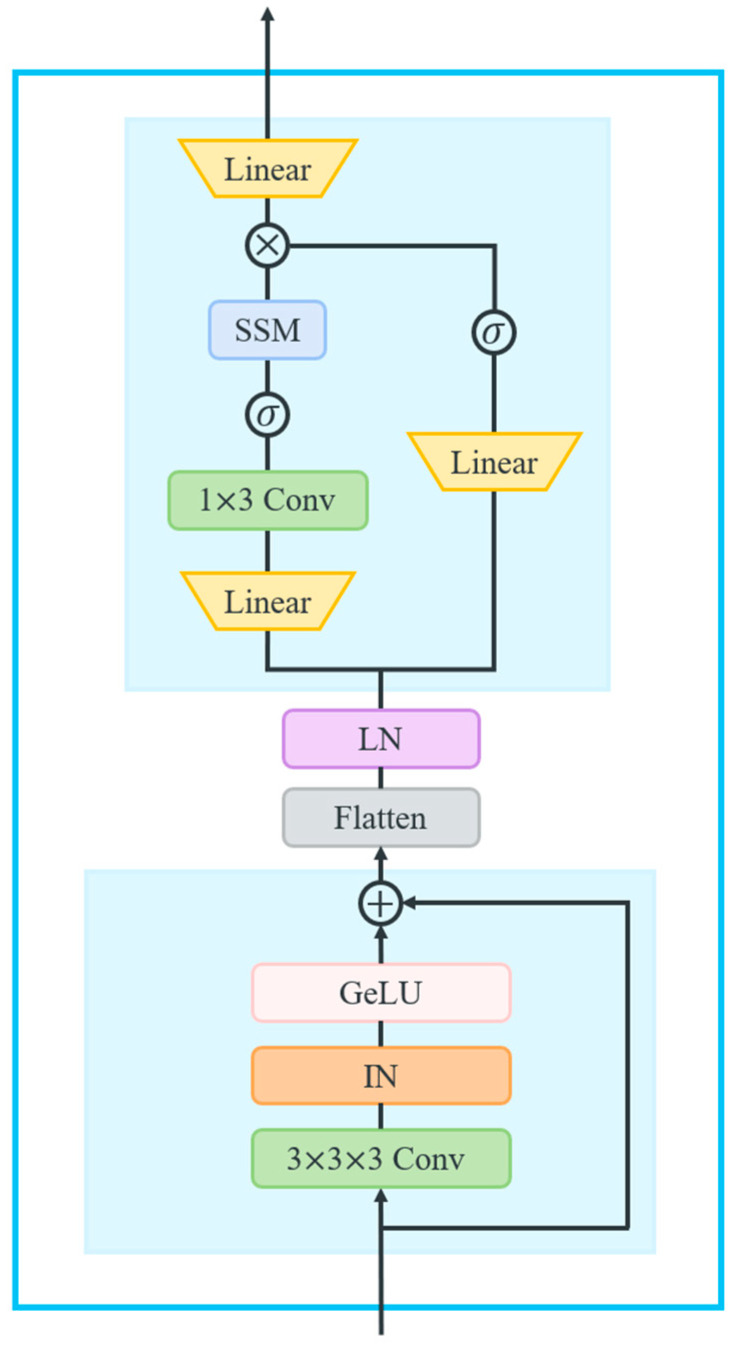
The operational principles of the External Mamba block.

The External Mamba block is designed based on the concept of selective state spaces, integrating the State Space Models (SSMs) with selective attention mechanisms to effectively capture long-range dependencies. Figure 6 illustrates the operational principles underlying this architecture. It is structured to include two successive Residual blocks, followed by a Mamba block. The Residual block comprises a convolutional layer, Instance Normalization (IN), and GeLU activation, as delineated in the following equations:

(19) $$\bar{X}=GeLU(IN({Conv3d}_{k=3,s=1,p=1}\left(X\right)))$$

The input features, represented as $X\in R^{\left(b,c,\;t,\;h,\;w\right)}$, are first processed through the two Residual blocks. Subsequently, the output is flattened and transposed to $R^{\left(b,\;c,\;l\right)}$, where l=t×h×w. Following Layer Normalization, features enter the Mamba block, which comprises two parallel branches.

In the first branch, features are expanded to $R^{\left(b,\;2c,l\right)}$ through a linear layer and a 1D convolutional layer, followed by the SiLU activation function. This is combined with the SSM layer [37], which leverages a selective state space model to capture long-range dependencies. The core operations of this block involve discretizing the continuous system parameters (Δ, A, B) into the discrete parameters $\left(\bar{A},\;\bar{B}\right)$, as defined in Equations (20) and (21), and computing the output of the first branch $y_{t}^{1}\in R^{l}$ through a linear recurrence governed by Equations (22) and (23).

(20) $$\bar{A}={\;f}_{A}\left(\Delta ,\;A\right)=\exp\left(\Delta A\right)$$

(21) $$\bar{B}=\;f_{B}\left(\Delta ,\;A,\;B\right)={\left(\Delta A\right)}^{-1}\left(\exp\left(\Delta A\right)-I\right)\cdot \;\Delta B$$

(22) $$h_{t}^{\prime }=\bar{A}h_{t}+\bar{B}{\bar{x}}_{t}$$

(23) $$y_{t}^{1}=Ch_{t}^{\prime }$$

Next, the second branch similarly expands features to $R^{\left(b,\;2c,l\right)}$ using a linear layer and SiLU activation, yielding the output $y_{t}^{2}\in R^{l}$ at time t. Subsequently, the two branches’ outputs are combined using the Hadamard product, resulting in features projected back to $R^{\left(b,\;c,\;l\right)}$ and reshaped to $R^{\left(b,\;c,\;t,\;h,\;w\right)}$:

(24) $$y_{t}=y_{t}^{1}\;⨀{\;y}_{t}^{2}$$

where $y_{t}^{1}$, ${\;y}_{t}^{2}\in R^{l}$ represent the outputs of two branches at time t, respectively, and ⨀ denotes the Hadamard product.

Furthermore, reliance solely on the hidden state from the preceding time step in traditional LSTM networks may restrict the network’s ability to identify extensive dependencies, complex patterns, and unpredictable temporal relationships. This limitation can lead to error propagation, adversely affecting predictive accuracy over extended periods. To mitigate these challenges, we incorporate the hidden states from the two preceding time steps rather than just one, as illustrated by the following equations:

(25) $${\tilde{h}}_{t-1}=Concat\left(h_{t-1},\;h_{t-2}\right)$$

(26) $${\tilde{h}}_{t-1}=Linear\left(h_{t-1}\right)$$

where $h_{t-1}$ and $h_{t-2}$ denote the hidden states of the two preceding time steps prior to time t.

Similarly to standard LSTMs, each unit within the MASA-LSTM comprises three gates: the forget gate $f_{t}$, the input gate $i_{t}$, and the output gate $o_{t}$, which represent the values of their respective operations. Each gate consists of multiple hidden neurons responsible for filtering, updating, and outputting information. The calculation formulas for these gates are as follows:

(27) $$f_{t}=\sigma \left(W_{f}y_{t}+V_{f}{\tilde{h}}_{t-1}+b_{f}\right)$$

(28) $$i_{t}=\sigma \left(W_{i}y_{t}+V_{i}{\tilde{h}}_{t-1}+b_{i}\right)$$

(29) $$o_{t}=\sigma \left(W_{o}y_{t}+V_{o}{\tilde{h}}_{t-1}+b_{o}\right)$$

where $W\in R^{d\times l}$ and $V\in R^{d\times d}$ are the shared weight matrices and the value matrix, respectively, and $b\in R^{d}$ is the iteratively updated shared bias vector. The dimension of the hidden vectors is denoted as d, and the activation function is represented by σ. Then, the newly acquired information $s_{t\;}$ at time t can be obtained as follows:

(30) $$s_{t}=tanh\left(W_{s}y_{t}+V_{s}{\tilde{h}}_{t-1}+b_{s}\right)$$

The cell state $C_{t}$ serves as a repository for historical information, enabling the preservation of long-range dependencies. We introduce adaptive trainable parameters α and β as scaling factors in the computation of $C_{t}$, allowing for greater flexibility in controlling information flow during training. The calculation formula is expressed as follows:

(31) $$C_{t}=\alpha \times f_{t}⊗C_{t-1}+\beta \times i_{t}⊗s_{t}$$

Once $C_{t-1\;}$ is discarded and the information is updated, $s_{t\;}$ reverts back to $C_{t\;}$. At the output gate, ${\tilde{h}}_{t}$ is the value output from $C_{t\;}$, as illustrated in the following equations:

(32) $${\tilde{h}}_{t}=o_{t}⊗tanh\left(C_{t}\right)$$

Following each time step iteration, a multi-head self-attention mechanism is employed to capture local long-range dependencies [38]:

(33) $$Q={\tilde{h}}_{t}\cdot W_{i}^{Q},\;K={\tilde{h}}_{t}\cdot W_{i}^{K},\;V={\tilde{h}}_{t}\cdot W_{i}^{V}$$

(34) $$Attention\left(Q,\;K,\;V\right)=softmax\left(\frac{QK^{T}}{\sqrt{d_{k}}}\right)V$$

(35) $${head}_{i}=Attention\left(QW_{i}^{Q},\;KW_{i}^{K},VW_{i}^{V}\right),\;i=1,\;\ldots ,\;h,$$

(36) $$MSA\left({\tilde{h}}_{t}\right)=Concat\left({head}_{1},\ldots {head}_{h}\right)W_{i}^{O}$$

Finally, the hidden state $h_{t}$ is computed through the residual layer and layer normalization, and this state is subsequently input into the next time step for iterative computation, as follows:

(37) $$h_{t}=LN\left({\tilde{h}}_{t}+MSA\left({\tilde{h}}_{t}\right)\right)$$

Therefore, the comprehensive framework of the MASA-LSTM can enhance the model’s performance in capturing complex temporal dependencies within time series data.

## 3. Experiments

In this section, we conducted experiments to evaluate our proposed model by assessing its prediction accuracy and robustness under varying noise levels. We also compare our proposed model with other state-of-the-art deep learning models. Furthermore, we conducted ablation studies to evaluate the effectiveness of our proposed Mamba-enhanced adaptive self-attention LSTM, multi-channel denoised image input, and the proposed MOM-Conv layer.

### 3.1. Experimental Setup

This section outlines the details of the two datasets used in the experiments, as well as the experimental configuration and associated data preprocessing methodology. The experiments were carried out under stable operating conditions with the following hardware specifications: an NVIDIA GeForce RTX 3060 12 GB GPU (NVIDIA Corporation, Santa Clara, CA, USA) and a 12th Generation Intel(R) Core(TM) i5-12400F 2.50 GHz CPU (Intel Corporation, Santa Clara, CA, USA). PyTorch 1.12 is the deep learning framework applied in all the experiments.

#### 3.1.1. Dataset Description

Experiments and analyses were performed on two distinct sets of data to evaluate the diagnostic performance of our model. The two datasets are the bearing dataset provided by Case Western Reserve University (CWRU) [39] and the bearing dataset from Paderborn University (PU) [40], both of which contain extensive experimental data. These datasets encompass a wide range of operational conditions and fault types, making them well-suited for comprehensive model training and validation. By focusing on these two representative datasets, the study enables a thorough evaluation of the proposed method under varied failure scenarios, thereby strengthening its robustness.

**CWRU bearing diagnostic dataset:** The CWRU bearing diagnosis dataset is derived from fault diagnosis experiments performed on the 6205-2RS rolling bearing manufactured by SKF [39]. This involves subjecting the bearings to various operating conditions at a sampling frequency of 12 kHz. Vibration signals of each bearing were collected under four different motor loads. Failures were deliberately introduced using electro-discharge machining (EDM), which involved carefully machining small defects into the surfaces of the rolling elements and races to simulate real-world faults. Specifically, single point failures measuring 0.18 mm, 0.36 mm, and 0.54 mm were introduced at the ball, inner raceway, and outer raceway under all operational situations. Moreover, the dataset includes ten distinct failure categories for each operational state, along with testing information for standard rolling bearings. Each sample consists of a fixed-length signal segment containing 1024 consecutive data, and 400 samples were collected. Furthermore, the dataset was split into training, validation, and testing sets in a ratio of 7:2:1. Accordingly, for each bearing state and load condition, 280 training samples, 80 validation samples, and 40 testing samples were extracted. Table 1 provides a detailed composition of the dataset.

**PU bearing diagnostic dataset:** The Paderborn bearing dataset was proposed by the University of Paderborn in Germany in 2014. The focus of the experiment is on rolling bearings of type 6203 [40]. Bearing vibration signals were recorded at a rate of 64 kHz, with each sample lasting 4 s. By adjusting the speed of the pressure system to regulate the radial pressure applied to the bearing and the load torque, fault data has been obtained under four distinct operational conditions. Condition 0 is one of the four distinct operational settings in the experiment and this case study specifically focuses on the data collected under condition 0. In this condition, the rotational speed of the test bearing is set at 1500 RPM, a load torque of 0.7 Nm, and a radial force of 1000 N. Normal and human-made defects include inner and outer ring defects treated using various processing methods. During our experiments on the PU dataset, 420 training samples, 120 validation samples, and 60 testing samples were chosen for each of the 9 different states, maintaining the same 7:2:1 ratio among training, validation, and testing samples, as illustrates in Table 2.

#### 3.1.2. Implementation Details

This section explains in detail the experiments performed using two datasets related to bearing failures, involving operating conditions and the experimental setting. Samples were sampled through sliding windows with overlapping. Each sample collected from a vibration signal contains 1024 data points, which are then transformed to form an image of 32×32 pixels. All the models compared in the experiment were trained with the same training set, employing the SGD optimizer with a learning rate of $3\times 10^{-4}$, a batch size of 16, and adopting the cross-entropy loss function. The iterative training process was stopped only when the loss became stable, and each experiment was performed 10 times to determine the average accuracy. Within the internal structure of the proposed model, the number of experts in the Mixture of Experts (MoE) mechanism is set to 3 for each MOM-Conv layer. The output channel numbers in the MOM-CNN layers increment sequentially (16, 32, 64, 128, 256) to facilitate hierarchical and comprehensive feature extraction. In the MASA-LSTM, the state dimension of the Mamba module is configured to 128, with a local convolution width of 4 and a module expansion factor of 2. Additionally, the hidden state size of the self-attention mechanism is set to 64, with 8 head units. The sequence length and hidden state dimension of the LSTM network are configured to 5 and 64, respectively.

#### 3.1.3. Noisy Environment

**Figure 7:**
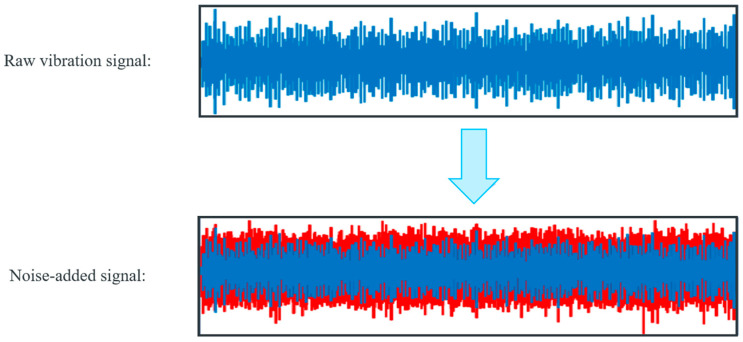
Signal after merging −10 dB Gaussian noise.

In practical manufacturing settings, capturing vibration signals while bearings are in operation can be disrupted by noise, potentially masking signs of faults. Figure 7 demonstrates how Gaussian noise is added to the raw bearing vibration signal, which can better simulate real-world operating environments. In addition, this approach has proven effective in suppressing model overfitting. Evaluation based on this noise-added signal can assess the anti-noise ability of fault diagnostic models. The signal-to-noise ratio (SNR) is commonly used to gauge the level of Gaussian noise present in a signal, defined as follows:

(38) $$SNR=10\;\times \;\log_{10}\left(\frac{P_{signal}}{P_{noise}}\right)$$

where $P_{noise}$ and $P_{signal}$ denote the power of the noise and the raw vibration signal, respectively.

### 3.2. Comparison with State-of-the-Art Methods

To illustrate the effectiveness, advancements, and robustness of our proposed model, particularly regarding its noise resistance and generalization capabilities, this study conducted a comparison with several widely used advanced benchmark methods. The subsequent sections provide a brief overview of several comparative models:

(1) The pyramid feature fusion network (PFFNet) [41] enhances rolling bearing fault diagnosis under small-sample conditions using multi-sensor data fusion and an expansion residual shrinkage weighting module. Its pyramid feature fusion network minimizes information loss, achieving robust accuracy across datasets.
(2) The Dual-path Multi-scale Attention Residual Network (DPMARN) [42] employs a dual-path structure with multi-scale convolutional branches and enhanced residual blocks with Squeeze-and-Excitation attention, enabling effective fault diagnosis in rolling bearings under noisy and variable load conditions.
(3) The TAR model [43] effectively integrates Transformer and ResNet18 architectures with a transfer learning strategy to enhance bearing fault diagnosis, leveraging a 1D convolutional layer for preprocessing, feature extraction, and robust classification, particularly in high-noise environments.
(4) The Swin Transformer [44] adapts to bearing fault diagnosis by treating time-frequency representations as images, effectively capturing local and global patterns through hierarchical feature extraction and self-attention, thus enhancing fault detection in complex signal data.
(5) WDCNN (Wide Deep Convolutional Neural Networks) [45] employs wide kernels for noise reduction and feature extraction, followed by smaller kernels for nonlinear mapping, using data augmentation and Adaptive Batch Normalization to enhance prediction accuracy in noisy and variable conditions.
(6) ResNet18 [46] is an 18-layer deep neural network that utilizes shortcut connections to learn residual functions, optimizing deeper architectures through a structure of convolutional layers and residual blocks, ultimately enabling efficient and effective performance in the task of bearing fault diagnosis.
(7) ResNeSt [47] improves fault diagnosis of rolling bearings by integrating channel-wise attention with multi-path representation through Split-Attention blocks, significantly enhancing feature representation and achieving state-of-the-art performance across various industrial applications and settings.

#### 3.2.1. Performance on the CWRU Bearing Dataset

**Figure 8:**
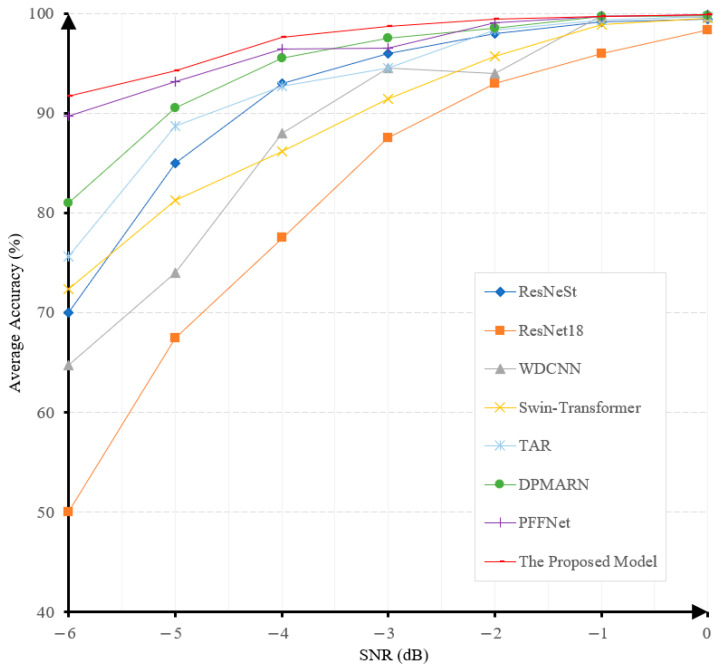
The prediction accuracies under different noise levels of various models on the CWRU dataset.

In this experiment, Gaussian noises with SNR between −6 and 0 dB are added to samples to evaluate the robustness levels of different models under noisy conditions. Figure 8 shows the accuracies achieved by different models at different noise levels.

The fault-diagnosis accuracy of all the algorithms exhibits a gradual decrease when the SNR decreases, as illustrated in Figure 8. It is noteworthy that our proposed model achieves the highest accuracy under all noise levels and the least performance degradation when the noise level increases. This demonstrates the superiority and robustness of our proposed model. The diagnostic accuracy of our model at the noise level of −6 dB is 91.8%, surpassing PFFNet [41], DPMARN [42], TAR [43], Swin-Transformer [44], ResNeSt [47], WDCNN [45], and ResNet18 [46] by large margins of 2.1%, 10.8%, 16.2%, 19.4%, 21.75%, 27.1%, and 41.8%, respectively. The robustness of our model to noise is mainly attributed to the use of the multi-channel denoising representation for input signals. This will also be verified in our ablation study.

**Figure 9:**
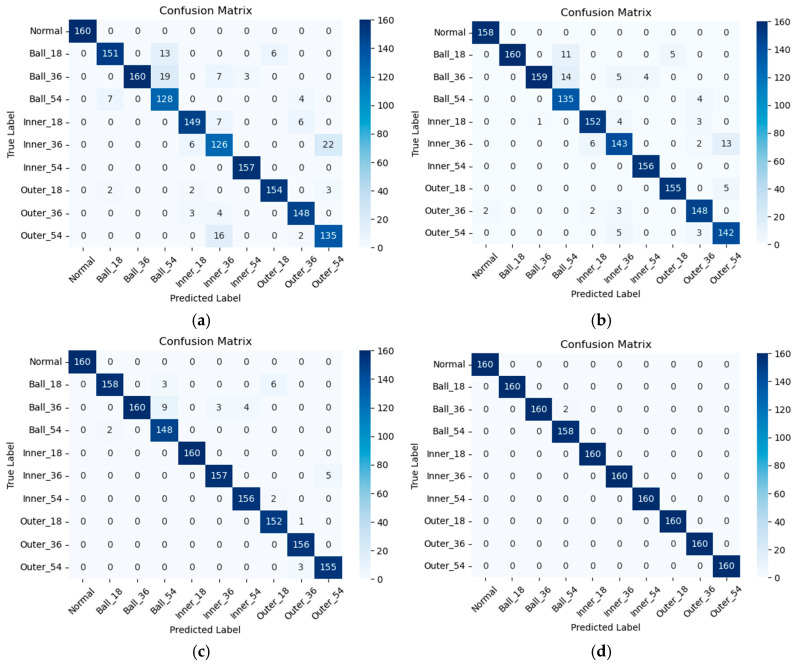
Confusion matrices of diagnostic results under different noise levels on the CWRU dataset: (**a**) −6 dB, (**b**) −5 dB, (**c**) −4 dB, and (**d**) 0 dB.

To understand the specific types of faults that the models are making, confusion matrices are also utilized to evaluate their performances. Figure 9 shows the classification performance of our proposed model on the CWRU dataset under different noise levels. Irrespective of the level of noise present in the environment, the proposed model consistently ensures that the predicted results maintain a notably high level of accuracy. As shown in Figure 9a, the proposed model achieved a prediction accuracy of 91.8% in a −6 dB noise setting. Furthermore, the prediction accuracy of the proposed model is able to achieve 99.9% in a 0 dB noise setting, as shown in Figure 9d. The research results obviously show that the proposed approach is able to accurately classify various faults of rolling bearings, showing excellent accuracy and reliability. However, in a high level of noise setting, the diagnostic outcomes of the proposed approach are less efficient for detecting 0.36 mm ball failure. Regardless, the total diagnostic accuracies remain at or above 90%.

**Figure 10:**
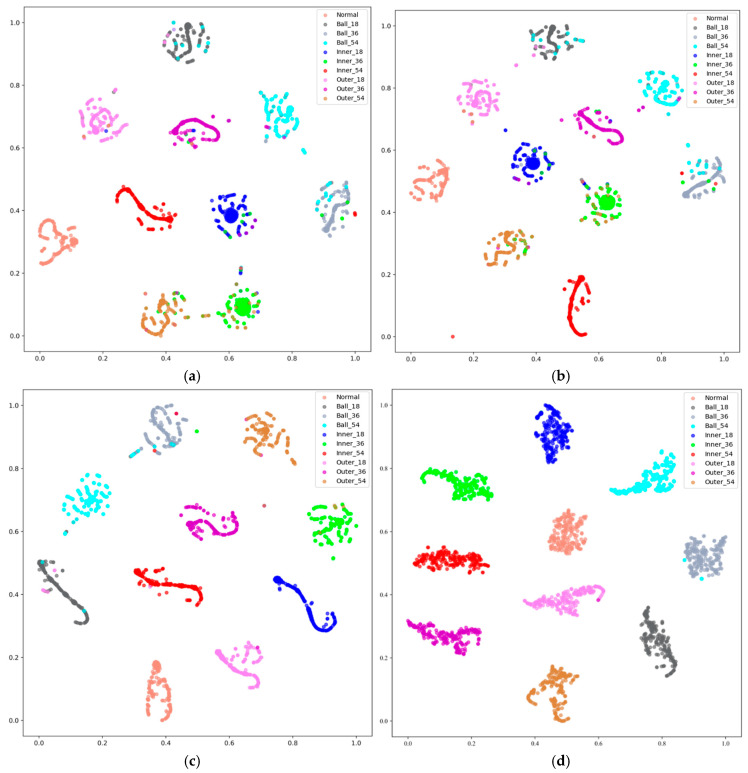
Analysis of feature clusters under different noise levels on the CWRU dataset: (**a**) −6 dB, (**b**) −5 dB, (**c**) −4 dB, and (**d**) 0 dB.

Furthermore, we also study the clustering of the samples belonging to different fault classes under different noise levels for our proposed model. T-SNE [48] is employed to reduce the dimensions of the features generated by the proposed model. Figure 10 provides the visualization results for the features produced on the CWRU test dataset. When the noise level increases, the spread of the different clusters changes slightly, but more outliers appear. Among these visualizations, more sample types overlap under −6 dB noise environment, while only several sample types overlap under the 0 dB noise environment. The clustering effect in the experiments is significantly enhanced as the value of SNR increases. This improvement demonstrates the superior clustering capability of our proposed model.

#### 3.2.2. Performance on the PU Bearing Dataset

The aim of this study is to demonstrate the excellent accuracy and noise resistance of the fault diagnostic model on the PU dataset in this section. In order to accomplish this objective, we evaluate the proposed model against various advanced benchmark fault diagnosis approaches in deep learning.

**Figure 11:**
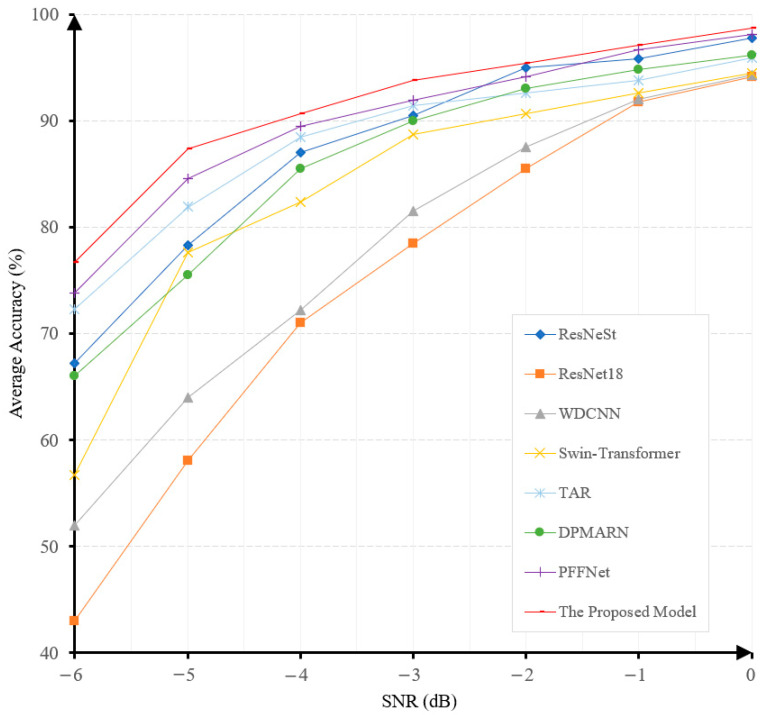
The prediction accuracies under different noise levels of various models on the PU dataset.

In the same way, Figure 11 shows the prediction accuracies of different methods for diagnosing bearing faults at different levels of noise. According to Figure 11, the proposed model demonstrates the highest diagnostic accuracy of 76.7% in the noisy setting (SNR = −6 dB), surpassing PFFNet [41], TAR [43], ResNeSt [47], DPMARN [42], Swin-Transformer [44], WDCNN [45], ResNet18 [46] by margins of 2.9%, 4.4%, 9.5%, 10.7%, 20%, 24.7%, and 33.7%, respectively. Furthermore, across all noise levels, the proposed method consistently achieves superior accuracies compared to other models. As the noise intensity decreases, its accuracy continues to improve and is always maintained above 75%. Through examination and comparison, it is clear that the proposed approach demonstrates outstanding and stable diagnostic effectiveness in challenging settings.

**Figure 12:**
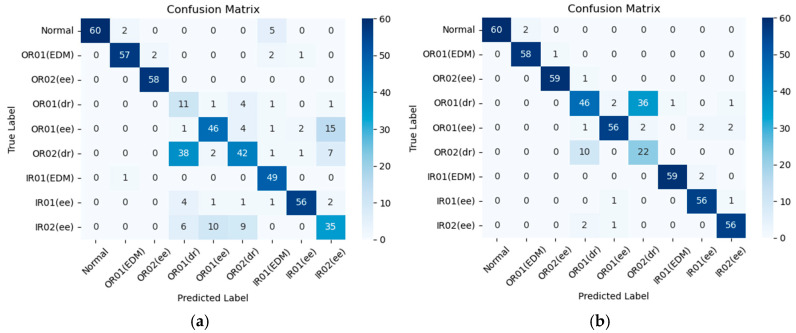

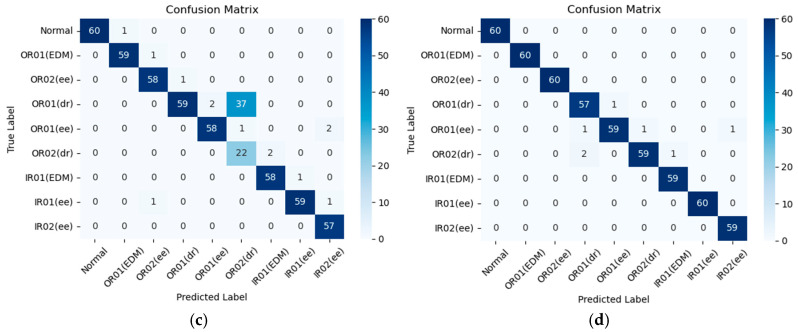
The confusion matrices of diagnostic results under different noise levels on the PU dataset: (**a**) −6 dB, (**b**) −5 dB, (**c**) −4 dB, and (**d**) 0 dB.

In addition, the confusion matrix was used for analysis. Based on the results in Figure 12a, our proposed model achieves a prediction accuracy of 76.7% in a −6 dB noise setting. Despite the noise level in the environment, the proposed model consistently maintains a relatively high standard of prediction accuracy, with an average accuracy exceeding 91% across all noise settings. As demonstrated in Figure 12b, the designed approach reaches a prediction accuracy of 87.4% in noisy conditions at −5 dB. Moreover, it also reaches a prediction accuracy of 90.7% and 98.7% in noisy conditions at −4 dB and 0 dB, as illustrated in Figure 12c,d. Overall, this proposed model demonstrates the amazing efficacy in accurately and reliably detecting various faults states of rolling bearings.

**Figure 13:**
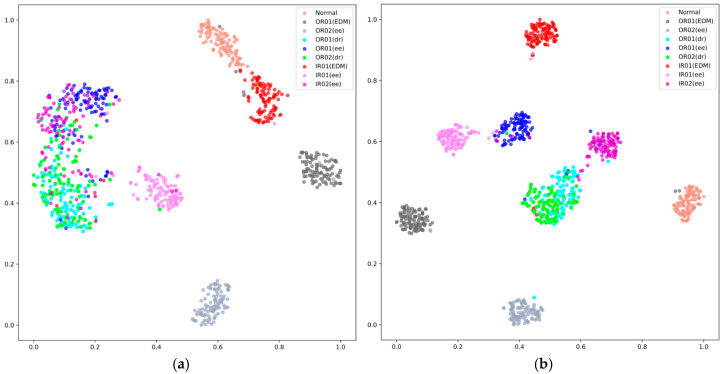

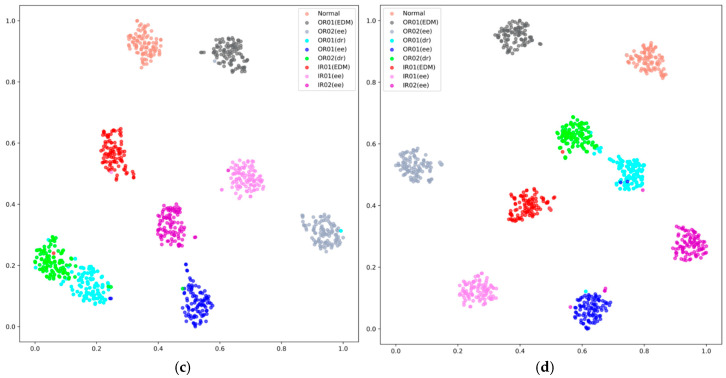
Analysis of feature clusters under different noise levels on the PU dataset: (**a**) −6 dB, (**b**) −5 dB, (**c**) −4 dB, and (**d**) 0 dB.

Similarly, applying t-SNE technology, the visualization of clustering results of our proposed model on the PU dataset are showcased in Figure 13. The visualization of feature vectors indicates that, under varying noise environments, the degree of overlap among different sample clusters varies when utilizing our model. Figure 13d indicates that in a noise-free environment, the overlap among different types of feature clusters is minimized, while in a −6 dB noise setting, as shown in Figure 13a, there is a significant overlap in sample types. The overall trend also indicates that as noise increases, the overlap between different types of feature clusters gradually intensifies. These findings also underscore the methodological advantages of our proposed model adopting the three methodologies, notably resulting in enhanced clustering capabilities.

### 3.3. Ablation Study

#### 3.3.1. The Impact of Multi-Channel Denoised Image Input Methodology

In this section, we not only investigated the impact of the multi-channel denoised image input methodology on the predictive performance of our proposed model but also separately examined the influence of each of the three channels—original, mean, and median—within the current three-channel denoised image input methodology on the proposed model’s final prediction results. Therefore, five sets of experiments were designed for comparison, each performed under varying levels of noise. These experiments consisted of five configurations and are outlined below:

- “w/o three-channel”: The proposed model no longer adopts the three-channel denoised image input methodology.
- “w/o original-channel”: This configuration excludes the original-channel from the three-channel denoised image input methodology.
- “w/o mean-channel”: This configuration excludes the mean-channel from the three-channel denoised image input methodology.
- “w/o median-channel”: This configuration excludes the median-channel from the three-channel denoised image input methodology.
- “w/three-channel”: The proposed model adopts the three-channel denoised image input methodology.

The models used in all experiments retained the architecture of MOM-CNN and the Mamba-enhanced adaptive self-attention LSTM. All experiments were operated using the CWRU dataset. The resultant outcomes are depicted in Table 3.

Based on the prediction results presented in Table 3, we observe that the model exhibits the lowest accuracy without using any denoised channels at various signal-to-noise ratio (SNR) levels. Conversely, when employing the complete three-channel denoised image input methodology, the model achieves the highest average accuracy across all SNR levels, underscoring the importance of this approach in enhancing model performance. This further emphasizes the effectiveness of multi-channel denoising strategies and their critical role in improving the model’s noise resilience and overall performance. Additionally, by systematically removing each channel (original, mean, and median), we find that the original channel appears to contribute the most to the model’s performance, as indicated by the lowest accuracy in the “w/o original-channel” configuration. The mean channel follows, with its accuracy slightly higher than that observed when the original channel is removed. Lastly, the median channel has a relatively minor impact on the overall accuracy improvement.

#### 3.3.2. The Impact of Mixture of Multi-View Convolutional Layer

This section mainly focuses on studying the influence of integrating the MOM-Conv layer within the proposed model. Two sets of experiments were operated for comparative analysis, each operated across different noise levels. The experiments consisted of two configurations: ‘Without MOM-Conv layer’ and ‘With MOM-Conv layer’. All experiments applied 3-channel transformed images as input and integrated the Mamba-enhanced adaptive self-attention LSTM network. The experiments were based on the CWRU dataset, and the outcomes are illustrated in Table 4.

In the scenario with a −6 dB noise level, the model applying the MOM-Conv layer achieves a prediction accuracy of 91.8%, while the model without the MOM-Conv layer achieves only 84.2%. Thus, the inclusion of the MOM-Conv layer leads to a 7.6% increase in prediction accuracy. As the noise level increases, the trend persists whereby models integrating the MOM-Conv consistently achieve higher prediction accuracies than those without it. At a noise level of 0 dB, the model lacking the MOM-Conv layer achieves a prediction accuracy of 99.7%, whereas the model applying the MOM-Conv layer obtains a prediction accuracy of 99.9%. Therefore, integrating the MOM-Conv layer consistently yields superior performance compared to the other one without the MOM-Conv layer. This observation remains consistent across all varying noise environments, highlighting the beneficial impact of applying the MOM-Conv layer on prediction accuracy.

#### 3.3.3. The Impact of Mamba-Enhanced Adaptive Self-Attention LSTM

This section delves into the study of integrating the MASA-LSTM network within the proposed model. Two sets of experiments were operated to facilitate comparative analysis, each executed across various noise levels. These experiments comprised two configurations: ‘Without MASA-LSTM’ and ‘With MASA-LSTM’. All experiments employed 3-channel transformed images as input and integrated the MOM-Conv layer. The experiments were also operated on the CWRU dataset, and the resultant outcomes are elucidated in Table 5.

As the noise level increases, models incorporating the MASA-LSTM network consistently demonstrate higher prediction accuracies compared to those that do not utilize this integration. Specifically, in a −6 dB noise environment, the proposed model achieves a prediction accuracy of 91.8%, whereas the model lacking the MASA-LSTM integration attains only 83.6%. Furthermore, at a 0 dB noise level, the model without the MASA-LSTM integration reaches a prediction accuracy of 99.6%, in contrast to the model incorporating the MASA-LSTM network, which achieves an accuracy of 99.9%. Consequently, the integration of the MASA-LSTM network yields superior performance relative to models without this integration. This trend is observed across various noise environments, highlighting the beneficial impact of the MASA-LSTM network on prediction accuracy.

Additionally, the impact of each subcomponent of the MASA-LSTM on overall model performance has been studied. A total of five experimental setups were designed, with each experiment conducted under various noise environments to demonstrate the individual effectiveness of each component. The configurations of these five experiments are described as follows:

- “w/o external Mamba block”: The MASA-LSTM network omits the external Mamba block.
- “w/o multi-step state fusion”: This configuration removes the multi-step state fusion mechanism from the MASA-LSTM network.
- “w/o adaptive parameter tuning”: This configuration excludes the adaptive parameter tuning from the MASA-LSTM network.
- “w/o self-attention mechanism”: This configuration disables the self-attention mechanism.
- “w/all subcomponents”: The MASA-LSTM network incorporates all subcomponents.

All models in these experiments utilized the multi-channel denoised image input method and the MOM-CNN framework. The experiments were conducted using the CWRU dataset, and the results are presented in Table 6.

According to the results in Table 6, the model utilizing all subcomponents of MASA-LSTM achieves the highest accuracy, while the absence of any single subcomponent leads to a decline in performance. For example, removing the external Mamba block or the self-attention mechanism has the most significant impact, reducing accuracy to 88.3% and 87.8% under −6 dB noise conditions, respectively. These findings indicate that both components play a critical role in capturing long-range dependencies and global features. The absence of multi-step state fusion and adaptive parameter tuning also results in decreased accuracy, dropping to 89.5% and 90.2%, respectively, highlighting their importance in enhancing dynamic adaptability and information flow. Overall, the synergistic operation of all MASA-LSTM subcomponents significantly improves the model’s robustness and diagnostic accuracy in high-noise environments.

### 3.4. Hyperparameter Sensitivity Analysis

The performance of the proposed model is significantly influenced by the settings of its hyperparameters. During both the construction and training phases, several adjustable parameters play a crucial role. To assess their impact on model performance, we conducted a parameter sensitivity analysis. This section discusses the hyperparameters associated with both the MOM-CNN and MASA-LSTM networks.

#### 3.4.1. Sensitivity Analysis of MOM-CNN

**Figure 14:**
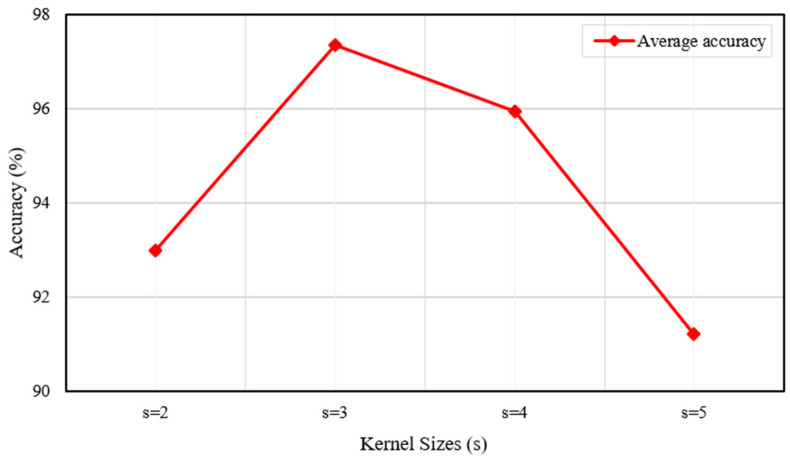
Average accuracy under various kernel sizes.

In the MOM-CNN framework, two key adjustable hyperparameters are the kernel size s and the number of experts k within the Mixture of Experts (MoE) mechanism. Regarding the kernel size, a larger value enables the model to capture a broader local region but may also result in the loss of detailed information. Therefore, simply increasing the kernel size does not necessarily improve model performance. During the training phase, we conducted comparative experiments using kernel sizes of {2, 3, 4, 5}, while keeping k fixed at 3. To accurately simulate hyperparameter selection under varying noise environments, which are typical in practical applications, we calculated the average accuracy of the model across all noise conditions (from −6 dB to 0 dB) and plotted its relationship with kernel size, as depicted in Figure 14. The results show that model accuracy exhibits an initial increase followed by a decrease as s varies across the specified range. The MOM-CNN framework achieves its optimal and most stable performance when s = 3.

**Figure 15:**
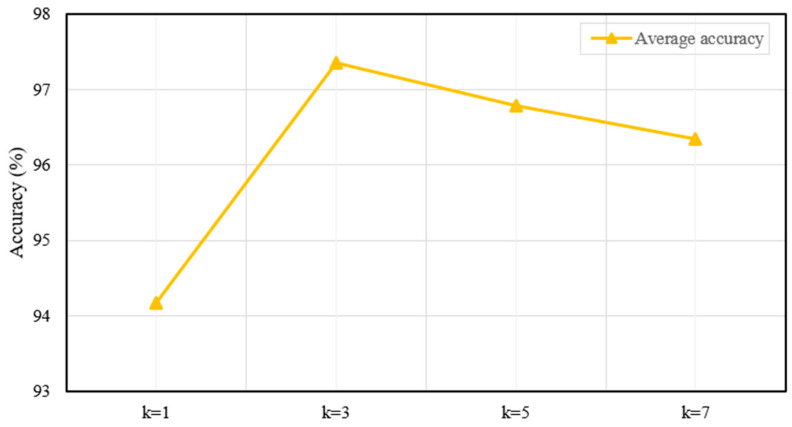
Average accuracy with different numbers of experts.

Similarly, appropriately increasing the number of experts enhances the model’s flexibility, as each expert can specialize in specific features or patterns, thereby improving overall prediction accuracy. To analyze the impact of varying k, we fixed the kernel size s at 3 and conducted experiments with different values of k. Figure 15 illustrates the accuracy for each case when k∈{1,3,5,7}. Based on the results, k=3 was selected as the optimal value.

The results reveal that as the number of experts increases, the model’s average accuracy gradually improves, peaking at 97.35% when k=3. This indicates that while an appropriate number of experts is crucial for optimal performance, exceeding this optimum yields diminishing returns.

#### 3.4.2. Sensitivity Analysis of MASA-LSTM Network

**Figure 16:**
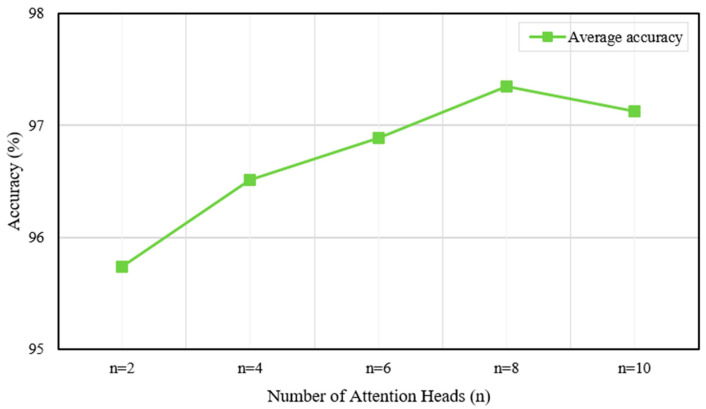
Average accuracy under different numbers of attention heads.

**Figure 17:**
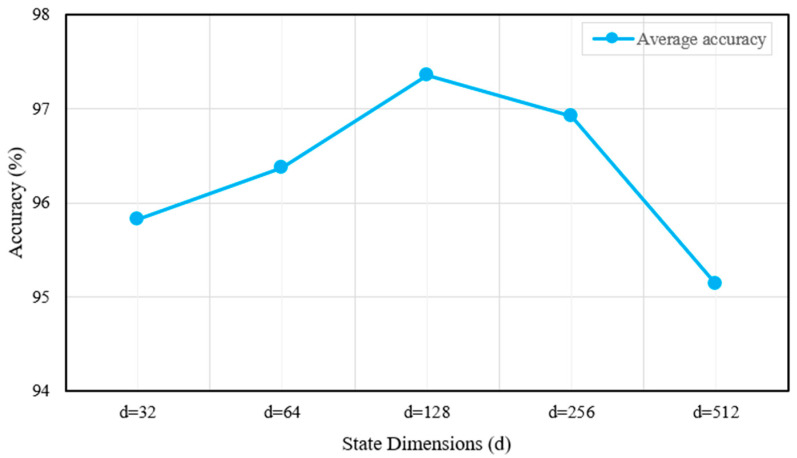
Average accuracy with different state dimensions.

In the MASA-LSTM framework, two additional critical hyperparameters are the number of attention heads n and the state dimension d of the Mamba module. To conduct a sensitivity analysis, we performed comparative experiments. First, with d fixed at 128, we varied n across {2, 4, 6, 8, 10}, as shown in Figure 16. Then, with n fixed at 8, we varied d across {32, 64, 128, 256, 512}. The corresponding results are presented in Figure 17.

As illustrated in Figure 16, the average accuracy improves with an increasing number of attention heads, reaching a peak value with eight heads. This indicates that a sufficient number of attention heads enables the model to capture more complex dependencies within the data, thereby enhancing performance. However, increasing n from 8 to 10 results in a slight decrease in accuracy, implying the introduction of redundancy or noise rather than valuable information.

Figure 17 indicates that the model achieves its highest accuracy when the state dimension is set to 128. However, further increases in the state dimension lead to a decline in accuracy. This observation demonstrates that excessively large dimensions may result in overfitting or unnecessary model complexity, ultimately degrading overall performance.

In summary, for the proposed hybrid network, the kernel size and number of experts in MOM-CNN, as well as the number of attention heads and state dimensions in MASA-LSTM, can be adjusted to achieve optimal performance, as discussed above.

### 3.5. Complexity Evaluation

To evaluate the model complexity of the proposed method, we compared its number of parameters, FLOPs, inference time, and accuracy with those of the baseline CNN-LSTM hybrid model. The comparison results are shown in Table 7.

In the complexity evaluation, from a trade-off perspective, our model exhibits a significant increase in both parameter count and floating-point operations compared to the baseline CNN-LSTM model (28.3% more parameters and 9.2% more FLOPs). Although these increases imply higher computational demands and a slight extension in inference time (51.23 s for our model versus 48.69 s for the baseline), the enhanced model complexity significantly improves accuracy, achieving 97.4%, well above the baseline model’s 86.9%. Therefore, while there is a modest sacrifice in computational resources and inference time, the performance improvement makes the model highly valuable for practical applications.

## 4. Conclusions

In this study, we propose a novel method for converting 1D signals into 2D images called the Multi-channel Denoised Image Input Method, along with an ensemble deep learning framework that integrates the Mixture of Multi-view Convolutional Neural Network (MOM-CNN) and the Mamba-enhanced Adaptive Self-Attention Long Short-Term Memory (MASA-LSTM) network. This approach addresses key challenges in feature extraction, environmental noise, and long-range dependency modeling. The MOM-Conv layer significantly enhances feature extraction and representation by effectively capturing and fusing local and contextual information, thereby improving prediction accuracy. Meanwhile, the MASA-LSTM model adeptly captures both global and local long-range dependencies from time series data, addressing the limitations of conventional models in extracting long-range dependencies in extremely long sequences. Additionally, the architecture incorporates adaptive parameter tuning and a multi-step state fusion mechanism, enabling flexible adjustments and more effective learning of temporal dependencies. The conversion of one-dimensional vibration signals into multi-channel denoised images further improves input representation and reduces noise interference, enhancing model stability and predictive capabilities. Our results demonstrate that the proposed model substantially outperforms existing state-of-the-art techniques on the Case Western Reserve University (CWRU) and Paderborn University (PU) bearing datasets, confirming its robustness and effectiveness across varying noise conditions. For instance, even in extremely noisy environments such as −6 dB, our model still achieves an accuracy of 91.8% on the CWRU dataset, surpassing other models by margins ranging from 2.1% to 41.8%. This work advances scientific understanding by introducing a novel signal-to-image conversion paradigm and a hybrid deep learning architecture that enhances noise-resilient, dependency-aware fault diagnosis. It offers potential to reduce unplanned downtime, enhance operational safety, and lower maintenance costs in industries reliant on rotating machinery.

However, the model also faces some limitations. Despite its strong performance across various noise environments, this improvement comes with increased model complexity and computational demands. Addressing this issue will be a focus of future research. Additionally, we plan to extend the application of our model to other rotating components, such as gears and pumps, to systematically validate and enhance its generalizability and robustness across diverse industrial tasks.

## Figures and Tables

**Table 1:** The composition of the rolling bearing dataset from CWRU.

Fault Type	Load (HP)	Diameter (mm)	Label	Tra/Val/Tes
OF	0/1/2/3	0.18	Outer_18	1120/320/160
	0/1/2/3	0.36	Outer_36	1120/320/160
	0/1/2/3	0.54	Outer_54	1120/320/160
IF	0/1/2/3	0.18	Inner_18	1120/320/160
	0/1/2/3	0.36	Inner_36	1120/320/160
	0/1/2/3	0.54	Inner_54	1120/320/160
BF	0/1/2/3	0.18	Ball_18	1120/320/160
	0/1/2/3	0.36	Ball_36	1120/320/160
	0/1/2/3	0.54	Ball_54	1120/320/160
Normal	0/1/2/3	-	Normal	1120/320/160

**Table 2:** Categories of actual fault information from the PU rolling bearing dataset.

Fault Type	Damage Type	Damage Level	Label	Tra/Val/Tes
IF	Electric etching	1	IR01 (ee)	420/120/60
	Electric etching	2	IR02 (ee)	420/120/60
	Electric discharge etching	1	IR01 (EDM)	420/120/60
OF	Electric etching	1	OR01 (ee)	420/120/60
	Electric etching	2	OR02 (ee)	420/120/60
	Drilling	1	OR01 (dr)	420/120/60
	Drilling	2	OR02 (dr)	420/120/60
	Electric discharge etching	1	OR01 (EDM)	420/120/60
Normal	-	-	Normal	420/120/60

**Table 3:** The average accuracies of proposed model with different number of channels (%).

Models	SNR (dB)						
	−6	−5	−4	−3	−2	−1	0
w/o three-channel	85.7	89.2	92.4	94.8	96.9	98.1	99.2
w/o original-channel	87.6	92.7	95.1	95.8	97.5	99.2	99.7
w/o mean-channel	88.4	92.1	95.8	96.4	97.4	99.5	99.9
w/o median-channel	90.8	93.5	95.6	97.2	98.1	99.4	99.9
w/three-channel	91.8	94.3	97.6	98.7	99.4	99.8	99.9

**Table 4:** The average accuracies of proposed model with or without MOM-Conv layer (%).

Models	SNR (dB)						
	−6	−5	−4	−3	−2	−1	0
Without MOM-Conv	84.2	91.8	95.2	97.9	98.7	99.4	99.7
With MOM-Conv	91.8	94.3	97.6	98.7	99.4	99.8	99.9

**Table 5:** The average accuracies of proposed model with MASA-LSTM or General LSTM (%).

Models	SNR (dB)						
	−6	−5	−4	−3	−2	−1	0
Without MASA-LSTM	83.6	90.2	96.4	97.9	99.1	99.4	99.6
With MASA-LSTM	91.8	94.3	97.6	98.7	99.4	99.8	99.9

**Table 6:** Average accuracies of the proposed model with various MASA-LSTM configurations (%).

Models	SNR (dB)						
	−6	−5	−4	−3	−2	−1	0
w/o external Mamba block	88.3	92.8	97.3	98.2	99.4	99.6	99.9
w/o multi-step state fusion	89.5	92.9	97.3	98.4	99.3	99.7	99.8
w/o adaptive parameter tuning	90.2	93.4	97.2	98.3	99.4	99.7	99.9
w/o self-attention mechanism	87.8	92.3	97	98.2	99.3	99.6	99.8
w/all subcomponents	91.8	94.3	97.6	98.7	99.4	99.8	99.9

**Table 7:** Experimental Results for Complexity Comparison.

Model	Parameters (M)	Flops (M)	Inference Time (s)	Accuracy (%)
Original CNN-LSTM	1.45	139.7	48.69	86.9
Our proposed model	1.86	152.5	51.23	97.4

## Data Availability

No new data were created or analyzed in this study. Data sharing is not applicable.
